# Accuracy of syndrome definitions based on diagnoses in physician claims

**DOI:** 10.1186/1471-2458-11-17

**Published:** 2011-01-07

**Authors:** Geneviève Cadieux, David L Buckeridge, André Jacques, Michael Libman, Nandini Dendukuri, Robyn Tamblyn

**Affiliations:** 1 Department of Epidemiology, Biostatistics and Occupational Health, McGill University, Montreal, Canada; 2 Direction de la Santé Publique de Montréal, Montreal, Canada; 3 Direction de l'amélioration de l'exercice, Collège des Médecins du Québec, Montreal, Canada; 4 Department of Medicine, McGill University, Montreal, Canada

## Abstract

**Background:**

Community clinics offer potential for timelier outbreak detection and monitoring than emergency departments. However, the accuracy of syndrome definitions used in surveillance has never been evaluated in community settings. This study's objective was to assess the accuracy of syndrome definitions based on diagnostic codes in physician claims for identifying 5 syndromes (fever, gastrointestinal, neurological, rash, and respiratory including influenza-like illness) in community clinics.

**Methods:**

We selected a random sample of 3,600 community-based primary care physicians who practiced in the fee-for-service system in the province of Quebec, Canada in 2005-2007. We randomly selected 10 visits per physician from their claims, stratifying on syndrome type and presence, diagnosis, and month. Double-blinded chart reviews were conducted by telephone with consenting physicians to obtain information on patient diagnoses for each sampled visit. The sensitivity, specificity, and positive predictive value (PPV) of physician claims were estimated by comparison to chart review.

**Results:**

1,098 (30.5%) physicians completed the chart review. A chart entry on the date of the corresponding claim was found for 10,529 (95.9%) visits. The sensitivity of syndrome definitions based on diagnostic codes in physician claims was low, ranging from 0.11 (fever) to 0.44 (respiratory), the specificity was high, and the PPV was moderate to high, ranging from 0.59 (fever) to 0.85 (respiratory). We found that rarely used diagnostic codes had a higher probability of being false-positives, and that more commonly used diagnostic codes had a higher PPV.

**Conclusions:**

Future research should identify physician, patient, and encounter characteristics associated with the accuracy of diagnostic codes in physician claims. This would enable public health to improve syndromic surveillance, either by focusing on physician claims whose diagnostic code is more likely to be accurate, or by using all physician claims and weighing each according to the likelihood that its diagnostic code is accurate.

## Background

Syndromic surveillance is used widely by public health departments to detect and monitor unusual disease activity in the population by extracting nonspecific clinical data from information systems in clinical settings [1-4]. Whereas much syndromic surveillance practice [3] and research [5] has focused on visits to emergency departments (ED), visits to community clinics offer another promising source of data. Syndromes followed in practice, such as influenza-like-illness (ILI), typically involve earlier, milder stages of disease, and most affected persons are likely to self-treat [6-8], at least initially, or present to walk-in clinics [6]. In fact, researchers have demonstrated that excess ILI activity can be detected earlier using data from clinics as compared to data from EDs [9-11]. The accuracy of diagnostic data from community clinics has not, however, been established.

Many syndromic surveillance systems use International Classification of Disease, 9^th ^revision (ICD-9) diagnostic codes in administrative databases to monitor syndrome occurrence [12]. For this purpose, expert panels have generated groupings of ICD-9 codes corresponding to conceptual syndrome definitions [13]. Administrative databases offer great promise for population-based surveillance by providing access to diagnostic information from many sites, including community healthcare settings. However, unlike medical procedure codes, ICD-9 diagnostic codes are not usually linked to healthcare provider payment, and therefore are not audited by health administrative authorities. Because of this, variation in diagnostic coding between physicians and between institutions is expected.

In a pilot study [14], we evaluated the accuracy of diagnostic codes in physician claims for identifying acute respiratory infections in nine Montreal-area community-based physicians. We abstracted the diagnosis from the medical chart for the 3,526 visits made by 729 sampled patients in 2002-2005, and compared the medical chart diagnosis to the ICD-9 code on the corresponding physician claim. For all acute respiratory infections combined, we found a sensitivity of 0.49, 95% CI (0.45, 0.53), and a positive predictive value (PPV) of 0.93, 95% CI (0.91, 0.94). These pilot study results are promising, but there is a need for a large-scale, population-based investigation of the accuracy of diagnostic codes used in syndromic surveillance.

The objective of the present study was to assess the accuracy of syndrome definitions based on diagnostic codes from a representative sample of physician claims for identifying 5 syndromes (fever, gastrointestinal, neurological, rash, and respiratory, including influenza-like illness (ILI)) in community healthcare settings. These syndromes were selected for their relevance to public health and the likelihood of being first detected among patients presenting to community healthcare settings.

## Methods

### Context

This study was conducted in the province of Quebec, Canada, where universal health coverage is provided through the provincial health insurance plan. Each Canadian province maintains a population-based registry of insured persons and claims for all physician visits remunerated on a fee-for-service basis. Physician claims include information on the diagnosis (recorded as an ICD-9 code), medical procedure, visit date, location, and cost of service. All claims also record unique physician and patient identifiers that can be used to create longitudinal histories of healthcare use. In the province of Quebec, 99% of residents have provincial health insurance and 85-95% of medical visits are remunerated on a fee-for-service basis [15]. In 2006, there were more than 7.6 million inhabitants in Quebec [16], and 18,908 active registered physicians [17]. The availability of diagnostic information for nearly all medical visits to Quebec physicians represents an invaluable opportunity for assessing the validity of using diagnostic codes in physician claims for population-based surveillance, including syndromic surveillance.

### Study design and sampling

**Figure 1:**
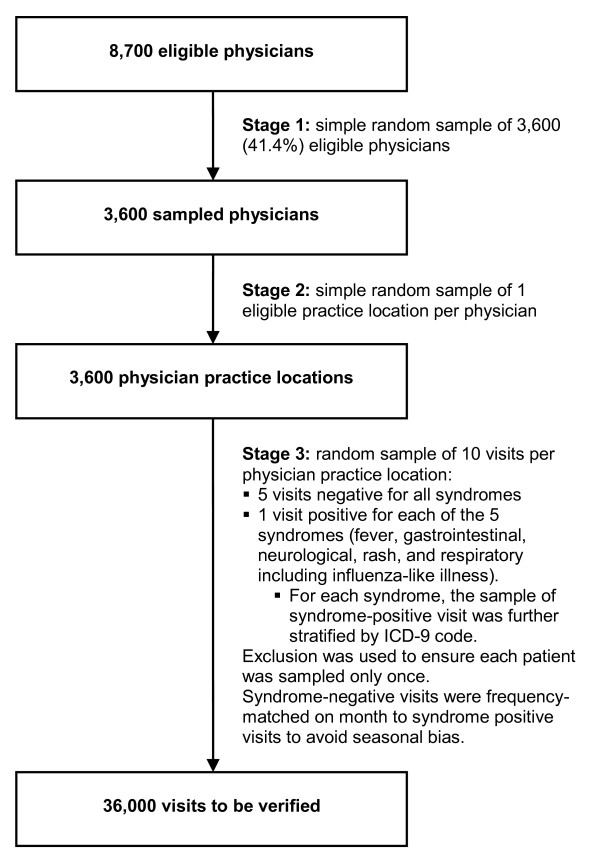
**Population-based, 3-stage stratified random sample of visits to all community physicians in the province of Quebec**.

The accuracy of diagnostic codes in physician claims for identifying syndromes was assessed by comparison to clinical information in the corresponding medical chart. To ensure representativeness, we used a population-based, 3-stage stratified random sample of 36,000 visits (Figure 1). In the first stage (Figure 1 Stage 1), the provincial health insurance agency identified all physicians who were eligible to be included in our study. To be eligible, physicians had to be a general practitioner, pediatrician, internist, geriatrician or general surgeon who practiced in the fee-for-service system in a private clinic, community health center, or hospital-based ambulatory care clinic during the 2-year study period (October 1, 2005 to September 30, 2007). Internists and general surgeons were included in our sample because, especially in rural-remote and underserved areas, these physicians may provide first-contact care and act as patients' family physician. From the 8,700 eligible physicians identified, the provincial health insurance agency selected a random sample of 3,600 (41.4%) physicians.

**Figure 2:**
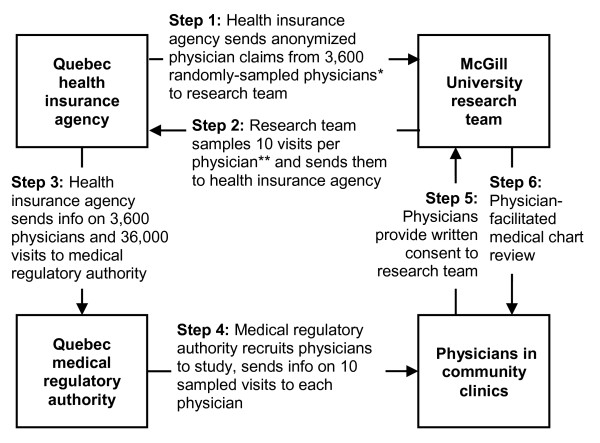
Overview of data collection * Physician sampling by the Quebec health insurance agency is described in Figure 1, Stages 1 and 2. ** Visit sampling by the research team is described in Figure 1, Stage 3.

In the second stage (Figure 1 Stage 2), to facilitate chart retrieval for review, the health insurance agency randomly selected one eligible community practice location for each physician. The health insurance agency then sent the research team an anonymized file containing all physician claims billed by the 3,600 physicians from their respective selected community practice location during the 2-year study period (Figure 2 Step 1).

In the third stage (Figure 1 Stage 3), the research team randomly selected 5 syndrome-positive visits, i.e., 1 visit for each of fever, gastrointestinal, neurological, rash, and respiratory syndrome (including ILI), and 5 visits negative for all syndromes. Visits were classified as positive for a syndrome if a physician claim for the visit had an ICD-9 code that was part of the syndrome definition. Because syndromes have low population prevalence, to maximize data collection efficiency [18], syndrome-positive visits were over-sampled relative to syndrome-negative ones, so as to yield 1 syndrome-positive visit per syndrome per physician and 5 syndrome-negative visits per physician. When sampling syndrome-positive claims, to maximize the number of syndrome-positive ICD-9 codes verified, we further stratified on ICD-9 code. Because two or more syndromes can occur concurrently in the same patient [19], syndrome-negative visits were negative for *all *syndromes. Syndrome-negative visits were also matched to syndrome-positive visits on calendar month to avoid bias due to syndrome seasonality. To avoid bias due to visits being clustered within patients, restriction was used to ensure that each patient was only sampled once. The list of 10 sampled visits was enumerated for each of the 3,600 physicians, for a total of 36,000 visits. An anonymized unique identifier, the study number, was assigned to each sampled visit by the research team. The list of 36,000 sampled visits was then sent to the health insurance agency (Figure 2 Step 2).

### Syndrome definitions

We verified two sets of definitions for the 5 syndromes under study: the definitions developed and published by the US Centers for Disease Control and Prevention (CDC) in 2003 [13], and used by the US Department of Defense's (DoD) Electronic Surveillance System for Early Notification of Community-based Epidemics (ESSENCE), as well as the corresponding definitions in the University of Pittsburgh's Real-time Outbreak and Disease Surveillance (RODS) system [19]. For ILI, we used the large-group (sensitive) and small-group (specific) definitions developed for the DoD ESSENCE system [20]. These definitions are similar to the consensus syndrome definitions being developed by representatives from the 10 syndromic surveillance systems in place in the US [21], which have not yet been mapped to ICD-9 codes.

### Physician recruitment

To preserve physician and patient anonymity, the health insurance agency sent the list of 3,600 physicians and 36,000 visits sampled by the research team to the medical regulatory authority (Figure 2 Step 3). The medical regulatory authority has the legal right to access confidential physician and patient information, therefore the list it received included physician names and mailing addresses, as well as patient names, insurance numbers, and dates of birth. The medical regulatory authority acted as a trusted third party and recruited physicians to the study on behalf of the research team; it also provided physicians with information on the 10 sampled visits (Figure 2 Step 4). Interested physicians mailed their written consent and contact information to the research team (Figure 2 Step 5). Non-responding physicians were sent up to four reminders. Physician recruitment began in September 2008 and ended in August 2009. To maximize participation, physicians were offered $50 compensation for their participation and a summary of study findings.

### Physician-facilitated medical chart review

The medical regulatory authority sent each physician the list of 10 sampled visits (Figure 2 Step 3). Lists sent to physicians included patients' first and last names, date of birth, health insurance number, and date of the visit to be verified, as well as the study number for each visit. Because the lists sent to physicians contained both patient information and study numbers, it enabled physicians to retrieve the relevant medical charts, and researchers to link the information collected through chart review to the anonymized physician claims file. During the chart review, interviewers and physicians referred to each visit using only the study number and visit date, thereby preserving patient anonymity.

Physician-facilitated medical chart reviews began in September 2008 and ended in December 2009. Using a previously published methodology [22], trained interviewers contacted consenting physicians by telephone to perform the chart review (Figure 2 Step 6). For each of the 10 sampled visits, the interviewer asked the physician to list all diagnoses. For each diagnosis corresponding to a syndrome definition, the interviewer asked the physician about the signs, symptoms, and key findings recorded in the medical chart, as well as the most likely etiology for the diagnosis (based solely on information available at the time of the visit).

Physician responses were entered directly into a database by the interviewer. Diagnoses were selected from a searchable list of diagnoses (mapped to ICD-9 codes) or, if the physician had recorded the ICD-9 code in the medical chart, the ICD-9 code was entered directly. For each syndrome-positive diagnosis, a list of syndrome-specific signs and symptoms was elicited, and the interviewer recorded whether the sign or symptom had been present, absent, or not recorded in the medical chart. Symptoms or signs not in the list and other key findings, such as epidemiologic links to other diagnosed cases or known outbreaks, were recorded as free text in separate fields. The data collection tool was translated to French for use with French-speaking physicians, and back-translated to English to ensure comparability of data collection.

At the time of chart review, the physician and interviewer were both blinded to the ICD-9 code in the physician claim and the syndrome-positive or syndrome-negative status of the claim. To minimize measurement error due to inter-rater differences, interviewers were trained to use the data collection tool. Inter-rater reliability was assessed at baseline by having interviewers perform 2 simulated physician interviews of 10 visits each (for a total of 20 visits). To maintain data quality, interviewers underwent quality assurance monitoring every 3 months. Each assessment was comprised of 2 simulated physician interviews of 10 visits each (for a total of 20 visits). Agreement between raters was measured using the intraclass correlation coefficient (ICC).

### Linkage of the medical chart review data to the physician claims data

The database containing the medical chart review data was linked to the physician claims file using the study number, physician identifier, and visit date. In our pilot study [14], we found that the visit date in the chart sometimes differed slightly from the visit date on the claim. We considered the chart and the claim to refer to the same visit if the visit date in the chart was within 0 (identical date) to 3 days from the visit date in the claim.

### Physician characteristics that may influence participation

Physician gender, preferred language (French or English), specialty, practice setting, and geographic location were obtained from the health insurance agency. Physician year of licensure was obtained from the medical regulatory authority. The number of days worked per year was calculated as the number of days when at least one claim was billed by the physician to the health insurance agency. The number of patients seen per day worked was calculated as the number of distinct patients for which one or more claim was billed by the physician per day worked. The number and prevalence of syndrome-positive visits were calculated for each physician using claims billed from the selected practice location during the 2-year study period.

### Statistical methods

For each visit, we assessed if the ICD-9 code in the physician claim and the diagnosis in the corresponding medical chart agreed as to the presence of each syndromes and ILI. For example, if the diagnosis in the claim was cough (786.2) and the diagnosis in the corresponding medical chart was acute bronchitis (466.0), then both the claim diagnosis and the chart diagnosis were positive for respiratory syndrome, therefore the claim was a true-positive for respiratory syndrome. If the diagnosis in the claim was cough (786.2) and the diagnoses in the chart were hypertension (401.9) and diabetes (250.0), then the claim diagnosis was positive for respiratory syndrome and the chart diagnoses were not, therefore the claim was a false-positive for respiratory syndrome.

The negative predictive value (NPV) of each syndrome definition was estimated directly from the data. Because we stratified syndrome-positive visits by ICD-9 code, we had to use an adjustment based on Bayes Theorem [23] to estimate the PPV of each syndrome. The PPV was estimated as a weighted average of each ICD-9 code's PPV, the weight being the number of visits with a given ICD-9 code divided by the total number of visits positive for that syndrome among participating physicians.

Because we verified more syndrome-positive visits than syndrome-negative ones, direct estimation of sensitivity and specificity using our data would lead to verification bias: sensitivity would be overestimated, and specificity underestimated [23]. Because verified claims were randomly sampled within syndrome-positive and syndrome-negative strata, unbiased estimation of these parameters was achieved by re-weighting for the verification fractions [23]. The sensitivity and specificity of physician claims for identifying each syndrome was estimated from the PPV and NPV [24] using the correction for verification bias [23], re-weighting for the different sampling fractions. We estimated the 95% CI for the bias-corrected sensitivity and specificity using the methods described by Begg and Greenes [23].

### Ethics review

The research protocol for this study was reviewed and approved by the McGill University Institutional Review Board, the Quebec privacy commission (Commission d'accès à l'information du Québec), the legal department of the Quebec health insurance agency (Régie de l'assurance maladie du Québec), and the Quebec medical regulatory authority (Collège des médecins du Québec).

## Results

Between October 1, 2005 and September 30, 2007, the 3,600 study physicians billed for over 20 million visits by 4.8 million patients (61% of the province's population) from their randomly selected community practice.

### Physician participation

Of 3,600 physicians contacted, 172 (4.8%) had an incorrect address on file with the health insurance agency, and 170 (4.7%) were discovered to be ineligible (recently deceased, retired, on sick/maternity leave, no longer practicing at the selected practice location). Of the 3,258 remaining physicians, 1,129 (34.7%) physicians consented to participate in the study, 218 (6.7%) refused, and 1,911 (58.7%) did not respond. Of the 1,129 consenting physicians, 1,098 (97.3%) completed the physician-facilitated medical chart review, and 31 (2.7%) were unreachable or withdrew consent prior to interview. Participating and non-participating physicians were similar on all measured variables except two (Table 1): as compared to non-participants, participants had been in practice longer and had worked more days during the study period. Syndrome prevalence was similar among participating and non-participating physicians, and ranged from 5 per 1,000 visits for neurological syndrome and ILI small-group, to 126 per 1,000 visits for respiratory syndrome.

**Table 1:** Characteristics of participating and non-participating physicians

	Participating physicians (N = 1,098)		Non-participating physicians (N = 2,160)	
**Physician characteristics**	**No**.	**(%)**	**No**.	**(%)**

Gender:				
Female	411	37.4	823	38.1
Male	687	62.6	1,337	61.9
Preferred language:				
French	1,006	91.6	1,937	89.7
English	92	8.4	223	10.3
Specialty:				
General practice	993	90.4	1,932	89.4
Internal medicine	13	1.2	41	1.9
Pediatrics	62	5.6	102	4.7
General surgery	30	2.7	85	3.9
Geriatrics	0	0	0	0
Type of setting selected:^1^				
Private clinic	1,060	96.5	2,044	94.6
Community health center	5	0.5	9	0.4
Hospital-based ambulatory clinic	33	3.0	107	5.0
Geographic location of selected setting:^1,3^				
Urban	921	83.9	1,867	86.4
Rural	177	16.1	293	13.6
	**Mean**	**SD**	**Mean**	**SD**
Years since licensure	24.2	9.7	22.3	10.5
No. days worked per year^1^	157.0	55.0	143.2	59.8
No. patients seen per day worked^1^	21.2	13.4	21.0	13.3

**Syndrome frequency based on claim ICD-9 code**	**No. visits**^1,2^	**Prevalence** **per 1,000 visits**^1^	**No. visits**^1,2^	**Prevalence** **per 1,000 visits**^1^

*CDC and DoD ESSENCE^4^*				
Fever	80,884	11	160,821	12
Gastrointestinal	162,282	22	309,209	24
Neurological	40,236	5	73,810	6
Rash	126,900	17	224,370	17
Respiratory	911,924	125	1,643,240	126
*RODS^5^*				
Fever	162,000	22	291,990	22
Gastrointestinal	146,355	20	283,578	22
Neurological	36,344	5	67,344	5
Rash	55,251	8	103,698	8
Respiratory	478,201	65	877,556	67
*Influenza-like illness^6^*				
Large-group	622,046	85	1,129,782	87
Small-group	32,173	4	61,127	5

^1^ As per our study design, for each physician, a single practice location was randomly selected to facilitate the validation process. The information in this table is based in claims generated from the selected practice location during the 2-year study period.

^2 ^There were a total of 7,315,994 visits to the 1,098 participating physicians, and 13,010,410 visits to the 2,160 eligible non-participating physicians at the selected practice location during the 2-year study period.

^3 ^We tested the statistical significance (at the p < 0.05 level) of any differences between participating and non-participating physicians using a multivariate logistic regression model where the dependent variable was participation and the independent variables were all characteristics in Table 1. Due to overlap between CDC, RODS, and ILI syndrome definitions, to avoid collinearity, we used separate models for each set of syndrome definitions. As compared to non-participating physicians, participating physicians had been in practice longer (odds ratio (OR)_per 10 years since licensure_, 1.15; 95% CI, 1.05-1.25), had worked more days (OR_per 50 days_, 1.18; 95% CI, 1.09-1.28) during the 2-year study period.

^4 ^Syndrome case definitions developed and published by the US Centers for Disease Control and Prevention (CDC) in 2003, and used by the US Department of Defense's Electronic Surveillance System for Early Notification of Community-based Epidemics (ESSENCE).

^5 ^Syndrome case definitions developed in the context of the University of Pittsburgh's Real-time Outbreak and Disease Surveillance (RODS) system.

^6 ^Syndrome case definitions developed in the context of the US Department of Defense's Electronic Surveillance System for Early Notification of Community-based Epidemics (ESSENCE).

### Inter-rater agreement

Agreement between raters was measured using simulated physician interviews shortly before the start of data collection and every 3 months thereafter. Agreement was perfect on all assessments (ICC = 1.00).

### Date agreement between the claim and the medical chart

Of the 10,980 visits selected for verification (10 visits per participating physician), physicians were able to access the corresponding medical chart for 10,669 (97.2%). The most common reasons for being unable to access the chart were inability to locate the medical chart (151 charts) and medical chart in storage with retrieval fee (140 charts). For 10,465 (98.1%) of the sampled visits, the visit date in the medical chart was identical to the visit date on the claim. Allowing for potential date transcription errors during billing, an additional 64 (0.6%) visits with a date in the medical chart that was within 1-3 days of the visit date on the claim were identified, for a total of 10,529 visits for which both the medical chart and the claim was available and the visit dates were in agreement (within the 3 day time window).

### Syndrome agreement between the claim ICD-9 code and the medical chart diagnosis

Table 2 shows the accuracy of ICD-9 codes in physician claims for identifying syndromes, as compared to the medical chart. The sensivity of ICD-9 codes in physician claims for identifying syndromes was low, ranging from 0.11, 95% CI (0.10, 0.13) for fever syndrome to 0.44, 95% CI (0.41, 0.47) for respiratory syndrome. The PPV of ICD-9 codes in physician claims for identifying syndromes was moderate to high, ranging from 0.59, 95% CI (0.55, 0.64) for fever syndrome to 0.85, 95% CI (0.83, 0.88) for respiratory syndrome. Both the specificity and NPV of ICD-9 codes in physician claims were near-perfect for all syndromes studied.

**Table 2:** Accuracy of ICD-9 coded diagnoses in physician claims, as compared to ICD-9 coded diagnoses from physician-facilitated medical chart review, for identifying constitutional, gastrointestinal, neurological, rash, and respiratory syndrome, as well as influenza-like illness (ILI) (N = 10,529 visits with matched claim-record pair)

Syndrome definition	**No**. visits in verified claims	**No**. visits in verified charts	Sensivity (95% CI)	Specificity (95% CI)	PPV (95% CI)	NPV (95% CI)
***CDC and DoD ESSENCE^1^***						
Fever	601	656	0.11 (0.10, 0.13)	0.99 (0.99, 0.99)	0.59 (0.55, 0.64)	0.94 (0.93, 0.95)
Gastrointestinal	855	888	0.23 (0.20, 0.26)	0.99 (0.99, 0.99)	0.71 (0.66, 0.75)	0.94 (0.94, 0.95)
Neurological	971	693	0.17 (0.14, 0.21)	1.00 (1.00, 1.00)	0.67 (0.64, 0.70)	0.98 (0.98, 0.98)
Rash	897	905	0.20 (0.18, 0.23)	0.99 (0.99, 0.99)	0.66 (0.62, 0.70)	0.95 (0.95, 0.96)
Respiratory	1,049	1,779	0.44 (0.41, 0.47)	0.97 (0.96, 0.98)	0.85 (0.83, 0.88)	0.84 (0.83, 0.85)

***RODS^2^***						
Fever	873	961	0.14 (0.12, 0.16)	0.99 (0.99, 0.99)	0.60 (0.56, 0.64)	0.91 (0.90, 0.92)
Gastrointestinal	703	834	0.20 (0.18, 0.23)	0.99 (0.99, 0.99)	0.68 (0.63, 0.73)	0.94 (0.94, 0.95)
Neurological	874	523	0.16 (0.13, 0.20)	1.00 (1.00, 1.00)	0.52 (0.48, 0.55)	0.99 (0.98, 0.99)
Rash	814	718	0.12 (0.10, 0.14)	1.00 (1.00, 1.00)	0.63 (0.59, 0.66)	0.96 (0.96, 0.97)
Respiratory	665	1,209	0.29 (0.26, 0.32)	0.98 (0.98, 0.98)	0.74 (0.70, 0.79)	0.87 (0.86, 0.88)

***Influenza-like illness^3^***						
Large-group	653	1,232	0.38 (0.35, 0.41)	0.98 (0.98, 0.98)	0.77 (0.73, 0.81)	0.88 (0.87, 0.89)
Small-group	53	49	0.18 (0.12, 0.26)	1.00 (1.00, 1.00)	0.29 (0.16, 0.41)	0.99 (0.99, 0.99)

^1 ^Syndrome case definitions developed and published by the US Centers for Disease Control and Prevention (CDC) in 2003, and used by the US Department of Defense's Electronic Surveillance System for Early Notification of Community-based Epidemics (ESSENCE).

^2 ^Syndrome case definitions developed in the context of the University of Pittsburgh's Real-time Outbreak and Disease Surveillance (RODS) system.

^3 ^Syndrome case definitions developed in the context of the US Department of Defense's Electronic Surveillance System for Early Notification of Community-based Epidemics (ESSENCE).

Additional file 1 (excerpted in Table 3) shows the PPV of physician claims for identifying syndromes for each ICD-9 code individually. There was wide variation in PPV between different ICD-9 codes in a given syndrome. ICD-9 codes that were very rarely used by physicians, for example tularemia (ICD-9 code: 21.9), had a high probability of being false-positives, and therefore a very low PPV. ICD-9 codes for common symptoms, for example fever (ICD-9 code: 780.6), had a lower probability of being false-positives, and a higher PPV. ICD-9 codes that represent common diagnoses, for example acute bronchitis (ICD-9 code: 466.0), had the lowest probability of being false-positives, and the highest PPV.

**Table 3:** Example of diagnostic codes with the highest and lowest positive predictive value (excerpted from additional file 1)

*Example of diagnostic codes with the HIGHEST positive predictive value (PPV)*			
**Syndrome**	**ICD-9 code**	**Diagnostic label**	**PPV (95% CI)**

Fever^1^	82.8	Tick-borne rickettsiosis not elsewhere classified	1.00 (1.00, 1.00)
	88.8	Other specified arthropod-borne diseases	1.00 (1.00, 1.00)

Gastrointestinal^1^	7.1	Giardiasis	1.00 (1.00, 1.00)
	5.9	Food poisoning not otherwise specified	1.00 (1.00, 1.00)

Neurological^1^	323.0	Encephalitis in viral disease classified elsewhere	1.00 (1.00, 1.00)
	784.3	Aphasia	1.00 (1.00, 1.00)

Rash^1^	53.8	Herpes zoster with unspecified complication	1.00 (1.00, 1.00)
	695.2	Erythema nodosum	1.00 (1.00, 1.00)

Respiratory^1^	33.0	*Bordetella pertussis*	1.00 (1.00, 1.00)
	462.9	Pharyngitis, acute not otherwise specified	1.00 (1.00, 1.00)

ILI large-group^2^	487.0	Influenza with pneumonia	1.00 (1.00, 1.00)
	486.0	Pneumonia, organism not otherwise specified	1.00 (1.00, 1.00)

***Example of diagnostic codes with the LOWEST positive predictive value (PPV)***			

**Syndrome**	**ICD-9 code**	**Diagnostic label**	**PPV (95% CI)**

Fever^1^	88.0	Bartonellosis	0.00 (0.00, 0.00)
	78.2	Sweating fever	0.00 (0.00, 0.00)

Gastrointestinal^1^	555.0	Regional enteritis, small intestine	0.00 (0.00, 0.00)
	1.1	Cholera due to *Vibrio cholerae *El Tor	0.00 (0.00, 0.00)

Neurological^1^	323.2	Encephalitis in protozoal disease classified elsewhere	0.00 (0.00, 0.00)
	53.0	Herpes zoster with meningitis	0.00 (0.00, 0.00)

Rash^1^	51.0	Cowpox	0.00 (0.00, 0.00)
	55.8	Measles complications not otherwise specified	0.00 (0.00, 0.00)

Respiratory^1^	20.4	Secondary pneumonic plague	0.00 (0.00, 0.00)
	79.8	Hantavirus infection	0.00 (0.00, 0.00)

ILI large-group^2^	490.0	Bronchitis not otherwise specified	0.00 (0.00, 0.00)
	465.8	Acute upper respiratory infection, other multiple sites	0.36 (0.08, 0.65)

^1 ^Syndrome case definitions developed and published by the US Centers for Disease Control and Prevention (CDC) in 2003, and used by the US Department of Defense's Electronic Surveillance System for Early Notification of Community-based Epidemics (ESSENCE).

^2 ^Syndrome case definition developed in the context of the US Department of Defense's Electronic Surveillance System for Early Notification of Community-based Epidemics (ESSENCE).

## Discussion

This study was the first large-scale, population-based investigation of the accuracy of syndrome definitions based on diagnostic codes in physician claims from community healthcare settings. We found that the sensitivity of syndrome definitions based on diagnostic codes in physician claims for identifying syndromes was low, the PPV was moderate to high, and the specificity and NPV were near-perfect. Even though our sensitivity estimates were low for all syndromes definitions, these syndrome definitions may still be useful for monitoring syndrome occurrence when there are large numbers of cases (e.g., seasonal influenza). Respiratory syndrome had the highest prevalence and was the most accurately reported in physician claims. Unexpectedly, ILI small-group had the lowest PPV of all syndromes definitions studied, much lower than previously reported by others [20]. The small-group definition of ILI is made up of only four ICD-9 codes: influenza with pneumonia (487.0), influenza with other respiratory manifestations (487.1), influenza with other manifestations (487.8), and acute upper respiratory infection, other multiple sites (465.8). Based on our interviews of over a thousand community physicians, we think that the poor accuracy of the ILI small-group definition reflects the common usage of the word 'flu' to describe a vague illness or a combination of non-specific symptoms. In addition to observing variation in physician claim accuracy between syndromes, we also found large variation in accuracy and prevalence between diagnostic codes within syndromes. Diagnostic codes with a very low prevalence were generally more likely to be false-positives; conversely, diagnostic codes with a higher prevalence were generally less likely to be false-positives, especially if they represented a diagnosis, as opposed to a symptom. This suggests that physicians are more likely to know the correct diagnostic code for a frequently diagnosed ailment, as compared to a rare one.

Rigorous attempts to assess the accuracy of ICD-9 codes used in syndromic surveillance as compared to the medical chart have been few, and they have relied on small convenience samples of emergency departments. In one such study, the accuracy of ICD-9 codes in ED reports for identifying 3 syndromes (fever, gastrointestinal, and respiratory) was assessed as compared to hospital chart diagnoses in the context of the US DoD ESSENCE surveillance system [25]. For greater data collection efficiency, syndrome-positive ED reports were over-sampled relative to syndrome-negative ones; however, analyses were not adjusted for this differential sampling strategy, resulting in verification bias [23], and leading to a large overestimation of sensivity and underestimation of specificity. To illustrate, the proportion of fever-positive visits in the sample was 0.19, whereas the proportion of fever-positive visits in the population is approximately 0.01 (based on our study). The authors reported a sensitivity of 0.69 and a specificity of 0.95. However, adjusting for verification bias, the estimates would be approximately 0.09 for sensivity and 1.00 for specificity, which is similar to our results. In another study, the accuracy of ICD-9 codes in ED reports for identifying 7 syndrome definitions (botulinic, constitutional, gastrointestinal, hemorrhagic, neurological, rash, and respiratory) was assessed against hospital chart diagnoses in the context of the RODS surveillance system [19]. To maximize the quantity of syndrome-positive ICD-9 codes verified, the investigators selected a random sample of syndrome-positive visits from ED reports, stratified on syndrome-positive ICD-9 code, such that an equal number of syndrome-positive visits was sampled for each ICD-9 code in a syndrome. For example, fever (780.6) and bubonic plague (020.0), both corresponding to constitutional syndrome, contributed the same number of cases. However, the prevalence and accuracy of each ICD-9 code in a syndrome is different, and because the analyses were not adjusted for the uniform sampling strategy used, the reported estimates of sensitivity, specificity, PPV and NPV are biased. In a third study [26], the accuracy of ICD-9 coded physician diagnoses from 9 hospital EDs for identifying 'acute respiratory illness' was assessed by comparison to medical chart review. A simple random sample was used; therefore the results were not subject to verification bias. The authors reported a sensitivity of 0.43, 95% CI (0.28-0.58) for acute respiratory illness, which is almost identical to our sensitivity estimate for respiratory syndrome; their estimates of NPV and specificity were also similar to ours, but their PPV estimate of 0.45, 95% CI (0.29-0.61) is much lower than ours.

Our study had several strengths and limitations. We used a large population-based random sample of all physicians working in the fee-for-service system in community healthcare settings in the province of Quebec in 2005-2007, thereby capturing potential ICD-9 coding differences between physicians, institutions, and regions. Not only did we estimate the accuracy of syndrome definitions, as others have done, but our study design enabled us to estimate the PPV of individual diagnostic codes within each syndrome definition. Matching syndrome-negative visits to syndrome-positive visit on calendar month ensured that our results were not affected by seasonal bias. Because two or more syndromes can occur concurrently in the same person [19], our requirement that syndrome-negative visits be negative for all syndromes ensured that we did not overestimate false-negatives and underestimate sensitivity and NPV. Our participation rate, though low, was consistent with that of other large population-based studies of Canadian physicians [27,28]. Participating and non-participating physicians were similar on nearly all measured variables. The participation rate was significantly lower among recently licensed physicians; recently licensed physicians may have been less likely to participate in our study because they tend to experience greater practice mobility [29] and report more impediments to practice [30] than their more experienced counterparts. Unfortunately, the accuracy of very rare syndrome-positive ICD-9 codes, such as cutaneous and pulmonary anthrax (22.0 and 22.1), could not be estimated because, as expected, they were not present in any of the 1,098 participating physicians' claims during the 2-year study period.

## Conclusions

We found that diagnostic codes in physician claims from community healthcare settings have low sensitivity, moderate to high PPV, and near-perfect specificity and NPV for identifying 5 syndromes (fever, gastrointestinal, neurological, rash, and respiratory, including ILI). Future research should evaluate the practical implications of our findings on decision-making in response to alerts from existing syndromic surveillance systems. Future research should also identify physician, patient, and encounter characteristics associated with better accuracy of diagnostic codes in physician claims. This would enable public health to improve syndromic surveillance, either by focusing on physician claims whose diagnostic code is more likely to be accurate, or by using all physician claims and weighing each according to the likelihood that its diagnostic code is accurate. We also estimated the prevalence and PPV of individual diagnostic codes within each syndrome. We found that rarely used diagnostic codes had a higher probability of being false-positives, and that more commonly used diagnostic codes had a higher PPV. These findings may be useful to the ongoing development of sensitive and specific consensus syndrome definitions, as either a sensitive or a specific definition may be more useful depending on the surveillance objective.

## Competing interests

The authors declare that they have no competing interests.

## Authors' contributions

All authors read and approved the final manuscript. GC collected the data, performed the data analysis, and is the primary author of the manuscript. AJ helped develop the methods and collect the data, and provided useful comments on the manuscript. ML helped develop the methods and provided useful comments on the manuscript. ND helped develop the methods, supervised the analysis, and provided useful comments on the manuscript. RT and DLB provided access to claims data and study subjects, helped develop the methods, oversaw the analysis, and provided useful comments on the manuscript.

## Pre-publication history

The pre-publication history for this paper can be accessed here:

http://www.biomedcentral.com/1471-2458/11/17/prepub

## Supplementary Material

Additional file 1 **Positive predictive value of individual ICD-9 codes within each syndrome case definition**. For all 12 syndrome case definitions investigated, the positive predictive value of diagnoses in physician claims is provided for each individual ICD-9 code. Click here for file

## References

[B1] Bravata DM Rahman MM Luong N Divan HA Cody SH A comparison of syndromic incidence data collected by triage nurses in Santa Clara County with regional infectious disease data J Urban Health 2003 80 2, Suppl 1 i122 Ref Type: Abstract 10.1007/BF02416907

[B2] Mandl KD Overhage JM Wagner MM Lober WB Sebastiani P Mostashari F Implementing syndromic surveillance: A practical guide informed by the early experience Journal of the American Medical Informatics Association 2004 11 141 150 10.1197/jamia.M1356 14633933 PMC353021

[B3] Buehler JW Sonricker A Paladini M Soper P Mostashari F Syndromic surveillance practice in the United States: findings from a survey of state, territorial, and selected local health departments Advances in Disease Surveillance 2008 6 1 8

[B4] Chretien JP Tomich NE Gaydos JC Kelley PW Real-Time Public Health Surveillance for Emergency Preparedness Am J Public Health 2009 99 1360 1363 10.2105/AJPH.2008.133926 19542047 PMC2707469

[B5] Buckeridge DL Outbreak detection through automated surveillance: A review of the determinants of detection J Biomed Inform 2007 40 370 379 10.1016/j.jbi.2006.09.003 17095301

[B6] Beaudeau P Payment P Bourderont D Mansotte F Boudhabay O Laubies B A time series study of anti-diarrheal drug sales and tap-water quality International Journal of Environmental Health Research 1999 9 293 311 10.1080/09603129973092

[B7] McIsaac WJ Levine N Goel V Visits by adults to family physicians for the common cold Journal of Family Practice 1998 47 366 369 9834772

[B8] Labrie J Self-care in the new millennium: American attitudes towards maintaining personal health 2001 Washington, DC, Consumer Health Care Products Association Ref Type: Report

[B9] Chan EH Tamblyn R Buckeridge D Timeliness of Ambulatory Data for Age-Specific ILI Surveillance Advances in Disease Surveillance 2008 5 1 10 Ref Type: Abstract

[B10] Chan E Evaluating the use of physician billing data for age and setting specific influenza surveillance 2009 McGill University; Master of Science

[B11] Greene S Kulldorff M Huang J Brand R Kleinman K Hsu J Timely Detection of Localized Excess Influenza Activity across Multiple Data Streams Advances in Disease Surveillance 2009 Ref Type: Abstract

[B12] Bravata DM McDonald KM Smith WM Rydzak C Szeto H Buckeridge DL Systematic review: Surveillance systems for early detection of bioterrorism-related diseases Annals of Internal Medicine 2004 140 910 922 15172906 10.7326/0003-4819-140-11-200406010-00013

[B13] Centers for Disease Control and Prevention Syndrome Definitions for Diseases Associated with Critical Bioterrorism-associated Agents 2003 1 29 Ref Type: Report

[B14] Cadieux G Tamblyn R Accuracy of Physician Billing Claims for Identifying Acute Respiratory Infections in Primary Care Health Services Research 2008 43 2223 2238 10.1111/j.1475-6773.2008.00873.x 18665858 PMC2614002

[B15] Regie de l'assurance-maladie du Quebec Statistiques Annuelles 2000 Regie de l'assurance-maladie du Quebec 46 48 Ref Type: Report

[B16] Statistics Canada Annual Demographic Estimates: Canada, Provinces and Territories - 2005-2006. 91-215-XIE 2006 Ottawa, Ontario, Statistics Canada 1 45 Ref Type: Report

[B17] Canadian Institute for Health Information Canada's Health Care Providers, 1997 to 2006, A Reference Guide 2008 Ottawa, Ontario, CIHI 1 188 Ref Type: Report

[B18] Irwig L Glasziou PP Berry G Chock C Mock P Simpson JM Efficient Study Designs to Assess the Accuracy of Screening-Tests Am J Epidemiol 1994 140 759 769 7942777 10.1093/oxfordjournals.aje.a117323

[B19] Chapman WW Dowling JN Wagner MM Generating a reliable reference standard set for syndromic case classification Journal of the American Medical Informatics Association 2005 12 618 629 10.1197/jamia.M1841 16049227 PMC1294033

[B20] Marsden-Haug N Foster VB Gould PL Elbert E Wang HL Pavlin JA Code-based syndromic surveillance for influenzalike illness by international classification of diseases, ninth revision Emerg Infect Dis 2007 13 207 216 10.3201/eid1302.060557 17479881 PMC2725845

[B21] Chapman WW Dowling JN Baer A Buckeridge DL Cochrane D Conway MA Developing syndrome definitions based on consensus and current use Journal of the American Medical Informatics Association 2010 17 595 601 10.1136/jamia.2010.003210 20819870 PMC2995670

[B22] Eguale T Tamblyn R Winslade N Buckeridge D Detection of Adverse Drug Events and Other Treatment Outcomes Using an Electronic Prescribing System Drug Saf 2008 31 1005 1016 10.2165/00002018-200831110-00005 18840020

[B23] Begg CB Greenes RA Assessment of Diagnostic-Tests When Disease Verification Is Subject to Selection Bias Biometrics 1983 39 207 215 10.2307/2530820 6871349

[B24] Kelly H Bull A Russo P McBryde ES Estimating sensitivity and specificity from positive predictive value, negative predictive value and prevalence: application to surveillance systems for hospital-acquired infections J Hosp Infect 2008 69 164 168 10.1016/j.jhin.2008.02.021 18448199

[B25] Betancourt JA Hakre S Polyak CS Pavlin JA Evaluation of ICD-9 codes for syndromic surveillance in the electronic surveillance system for the early notification of community-based epidemics Mil Med 2007 172 346 352 17484301 10.7205/milmed.172.4.346

[B26] Espino JU Wagner MM Accuracy of ICD-9-coded chief complaints and diagnoses for the detection of acute respiratory illness Proceedings of the AMIA Symposium 2001 164 168 PMC2243564 11833477

[B27] Grava-Gubins I Scott S Effects of various methodologic strategies Survey response rates among Canadian physicians and physicians-in-training Canadian Family Physician 2008 54 1424 1430 18854472 PMC2567275

[B28] Sullivan P Buske L Results from CMA's huge 1998 physician survey point to a dispirited profession Can Med Assoc J 1998 159 525 528 PMC1229656 9757182

[B29] Willke RJ Practice Mobility Among Young Physicians Medical Care 1991 29 977 988 10.1097/00005650-199110000-00004 1921530

[B30] Royal College of Physicians and Surgeons of Canada National Physician Survey Primer, October 2009: Generational Differences 2009 1 3 Ref Type: Report

